# Protection against insect predation during fruit development: the role of fleshy fruit wings of three species of *Zygophyllum* in the cold desert of Central Asia

**DOI:** 10.3389/fpls.2023.1267714

**Published:** 2023-11-14

**Authors:** Kaiqing Xie, Juan Qiu, Jannathan Mamut, Yuting Li, Dunyan Tan

**Affiliations:** ^1^Key Laboratory of Ministry of Education for Western Arid Region Grassland Resources and Ecology, College of Grassland Sciences, Xinjiang Agricultural University, Ürümqi, China; ^2^Xinjiang Key Laboratory for Ecological Adaptation and Evolution of Extreme Environment Biology, College of Life Sciences, Xinjiang Agricultural University, Ürümqi, China

**Keywords:** fruit wings, insect herbivory, protective function, seed development, seedling growth, *Zygophyllum*

## Abstract

**Introduction:**

Fruit wings serve various ecological functions, including facilitating wind dispersal, providing physical protection to seeds, and regulating seed germination. While many studies have reported the role of fruit wings in plants, little is known about their protective function during fruit development.

**Methods:**

In this study, winged fruits damaged by insects in natural populations of three *Zygophyllum* species (*Z. potaninii*, *Z. lehmannianum* and *Z. macropterum*) were investigated. We measured and compared the percentage of damaged winged fruits, seed set, seed mass, seed germination, and seedling growth of different insect herbivory categories.

**Results:**

The results revealed that the percentage of winged fruits with damaged wings only (low predation) was significantly higher than that of with damaged both fruit wings and fruit bodies (high predation). Furthermore, winged fruits with low predation had significant higher seed set, seed mass, seed germination, and dry mass and relative growth rate (RGR) in the seedlings which grown from the seeds, than that from winged fruits with high predation.

**Discussion:**

These results demonstrate that the presence of the fruit wings may provide protection for the seeds to alleviate harm from insect predation before dispersal. These findings provide new insights into the function of fruit wings and the reproductive strategies of desert plants.

## 1 Introduction

Fruit predation is a pervasive phenomenon experienced by plants across all biomes worldwide, severely limiting the recruitment and dynamics of many populations. It is an important ecological process affecting plant spatial distribution and species coexistence (Mezquida and Benkman, 2010; Van Klinken and White, 2014; Wang et al., 2019). Insect herbivory during fruit development can impact various reproductive traits, including the morphology of reproductive structures, fruit color, fruit and seed mass, seed viability, and seedling establishment (Janzen, 1971; Fedriani and Boulay, 2006; Meyer et al., 2014; Pardini et al., 2017). The interactions between phytophagous insects and their host plants are intrinsically complex and have evolved over a long period of co-evolution (Futuyma and Agrawal, 2009; Gedling et al., 2018; Ashra and Nair, 2022).

Accordingly, plants have developed a variety of physical barriers (e.g., thorns, trichomes, and cuticles), constitutive chemical mechanisms, and direct and indirect inducible defenses intended to reduce/counter the effects of insect herbivory (Kaplan et al., 2008; Wu and Baldwin, 2010; Mithöfer and Boland, 2012; War et al., 2012; Aljbory and Chen, 2018). In general, plants achieve protection for themselves primarily through their own accessory structures (Handley et al., 2005) and by attracting beneficial insects from additional trophic levels (Heil, 2008). For instance, trichomes can protect plants by decreasing insect oviposition (Handley et al., 2005), preventing larval movement (Verheggen et al., 2009; Figueiredo et al., 2013) or reducing larval feeding (Dalin and Björkman, 2003; Kariyat et al., 2017). Ant-plants (*Acacia drepanolobium*) typically provide hollow nest cavities and nutrition for the occupying ant colonies, and ants protect their host plants from herbivory (Madden and Young, 1992; Stanton et al., 1999).

Fruit wings are a common type of fruit appendage, and previous studies have primarily focused on their role in improving seed dispersal by wind (Wang and Wei, 2007; Yu et al., 2009) and water (Planchuelo et al., 2016; Tessier, 2019). In addition, several studies have explored their potential to inhibit seed germination through constitutive features (Wei et al., 2008; Yu et al., 2009) or the presence of specific natural chemicals (El-Keblawy et al., 2014; Bhatt et al., 2017). However, the function of fruit wings during fruit development has not been reported.

*Zygophyllum* L. (Zygophyllaceae) comprises approximately 150 species, distributed widely across arid desert regions of Africa, Europe, Asia and Australia (Liu and Zhou, 1998; Van Zyl and Marias, 1999; Beier et al., 2003). Some species of this genus are important components of plant communities in arid regions and have ecological significance in maintaining plant diversity and stability in desert ecosystems (Liu and Zhou, 1998). The three *Zygophyllum* species studied (*Z. potaninii*, *Z. lehmannianum* and *Z. macropterum*) are typical xerophytic species that occur in the desert regions of Central Asia (Liu and Zhou, 1998). The winged fruits of the three species are succulent with fleshy wings during fruit development. Our field observation found that the fleshy fruit wings of the three species experienced higher insect damage than the fruit body in natural populations, and this damage occurred during the period of fruit development. Based on the winged fruit part damaged by insects, the winged fruits were classified into the following categories: winged fruit with no damage (intact winged fruit), winged fruit with damaged wings only (low predation), and winged fruit with damaged both wings and fruit bodies (high predation). Thus, we hypothesized that fleshy fruit wings provide protection for the seeds to alleviate harm from insect predation before dispersal.

To test this hypothesis, we compared the percentage of damaged winged fruits, seed set, seed mass, seed germination, and seedling growth of different insect herbivory categories. Specifically, we addressed the following questions: (1) How prevalent is insect herbivory in natural populations of the three *Zygophyllum* species? (2) How are the different insect herbivory categories distributed among the winged fruits? And (3) What are the effects of insect herbivory on seed development, seed germination and seedling growth of the three *Zygophyllum* species?

## 2 Materials and methods

### 2.1 Study area and species description

The study area is located in the Junggar Desert (84°31′–90°03′E, 44°11′–46°20′N) of the Xinjiang Uygur Autonomous Region, China. This area belongs to an inland cold desert with a typical temperate continental climate. The mean annual temperature is 7.6°C, with mean monthly temperatures during the coldest month (January) and hottest month (July) recorded at -23.9°C and 32.8°C, respectively. The average annual precipitation (including rain and snow) is 186.8 mm and the annual potential evaporation is >2000 mm (Shi et al., 2006).

*Zygophyllum potaninii* and *Z. macropterum* are reported to be perennial spring-flowering species, while *Z. lehmannianum* is an annual (Liu and Zhou, 1998; Mamut et al., 2014). They are widely distributed in the Junggar Desert and play a pivotal role in maintaining the stability of the desert ecosystem by increasing the surface roughness of the soil. These three species have spreading stems that are much branched at the base, and their fruits have five wings (Liu and Zhou, 1998). The sampling locations for each species are listed in **Supplementary Table 1**.

### 2.2 Percentage of winged fruits with different insect herbivory categories in natural populations

During June 13–29, 2021, we randomly selected 30 individuals at the mature winged fruits stage (before dehiscence) from 11 natural populations (**Supplementary Table 1**) of the three *Zygophyllum* species. The winged fruits from each individual were classified into three categories of insect herbivory: intact winged fruit, low predation, and high predation. The percentage of each insect herbivory category was calculated as (N_i_/N_t_) × 100, where N_i_ is the number of winged fruits with the same damaged part, and N_t_ is the total number of winged fruits from each individual.

### 2.3 Effect of insect herbivory on seed set and seed mass

Mature fruits (before dehiscence) were collected from 11 natural populations of the three *Zygophyllum* species during June 13–29, 2021. They were then divided into different insect herbivory categories, and winged fruits from these categories were separately stored in paper bags under laboratory conditions of temperature and relative humidity (18–30°C, 20–30% RH) until used in the following experiments. To determine the effect of insect herbivory on seed set and seed mass, we carried out two experiments after natural air-drying winged fruits for 7 d. (1) Thirty winged fruits were randomly selected from each of three insect herbivory categories, and the number of seeds and aborted ovules per fruit was recorded to determine seed set. (2) Ten replicates of 10 seeds of winged fruits from each of three insect herbivory categories were weighed using an electronic balance (BS210S, Satorius Co., Goettungen, Germany), and differences among the different insect herbivory categories were compared.

### 2.4 Effect of insect herbivory on seed germination

The purpose of this experiment was to determine the effect of insect herbivory on seed germination of the three *Zygophyllum* species, and two experiments were conducted as follows. (1) To investigate germination responses of seeds, four replicates of 25 seeds from intact fresh mature fruits (mixed collection from the study populations) were incubated on two layers of Whatman No. 1 filter paper moistened with 2.5 ml of distilled water in 9-cm-diameter plastic Petri dishes in light (12 h daily photoperiod) and constant darkness (Petri dishes with seeds wrapped with aluminum foil) at daily (12/12 h) temperature regimes of 5/2°C, 20/10°C, 25/15°C, 30/15°C, 35/20°C and 40/25°C. The criterion for germination was emergence of radicle from the seed (Lu et al., 2020). Germination under light was examined daily for 28 d, and all germinated seeds were removed at each counting. Germination in darkness was determined at the end of the 28 d experiment. (2) The purpose of this experiment was to compare seed germination of each insect herbivory category under optimum germination conditions. The optimum germination conditions were chosen based on the results of the preceding experiment. During July 18–August 3, 2021, four replicates of 25 seeds from each insect herbivory category were incubated at 35/20°C (*Z. potaninii* and *Z. lehmannianum*) and 30/15°C (*Z. macropterum*) in darkness for 28 d, respectively.

### 2.5 Effect of insect herbivory on seedling growth

The purpose of this experiment was to compare early seedling growth among the three insect herbivory categories of the three *Zygophyllum* species. During July 21–August 5, 2021, four replicates of 25 seeds from each insect herbivory category were incubated at 35/20°C (*Z. potaninii* and *Z. lehmannianum*) and 30/15°C (*Z. macropterum*) in darkness. After one week (according to seed germination time), 10 germinated seeds were selected to determine the dry mass of the seedlings at the beginning of the experiment. Also, 10 other germinated seeds were transferred under green light to a new Petri dish and incubated at optimum conditions for 14 days, with distilled water added every 5 d (under green light). At the beginning and end of the experiment, seedlings were dried at 80°C for 48 h and weighed using an electronic balance. The seedling relative growth rate (RGR) was calculated using the formula: RGR = (W_2_ − W_1_)/t_2_ − t_1_, where W_1_ and W_2_ represent the dry mass of a seedling at the beginning (W_1_) and end (W_2_) of the experiment, and (t_2_ − t_1_) represents the time interval (14 d) between measurements.

### 2.6 Data analysis

All data were tested for normality and homogeneity of variance before performing a one-way ANOVA. If the data were not normally distributed or if the variances were not homogeneous, the non-parametric Kruskal-Wallis test was used. All data were expressed as the mean ± SE. Kruskal-Wallis test was used to determine the differences in percentage of different insect herbivory categories among the study populations and in the same population. One-way ANOVA was used to determine the differences in seed set and seed mass among different insect herbivory categories.

A generalized linear model (GLM) with binary logistic regression models with germination as a binomial response variable (e.g., germinated versus non-germinated) was used to test the significance of the main effects (temperature and light) and their interaction on germination. The same method was used to determine the differences in seed germination among different insect herbivory categories under optimal germination conditions. One-way ANOVA was used to determine the differences in seedling dry mass and seedling RGR among different insect herbivory categories. All statistical tests were conducted using IBM SPSS version 25.0 (SPSS Inc, Chicago, IL, USA).

## 3 Results

### 3.1 Percentage of winged fruits in different insect herbivory categories

**Figure 1:**
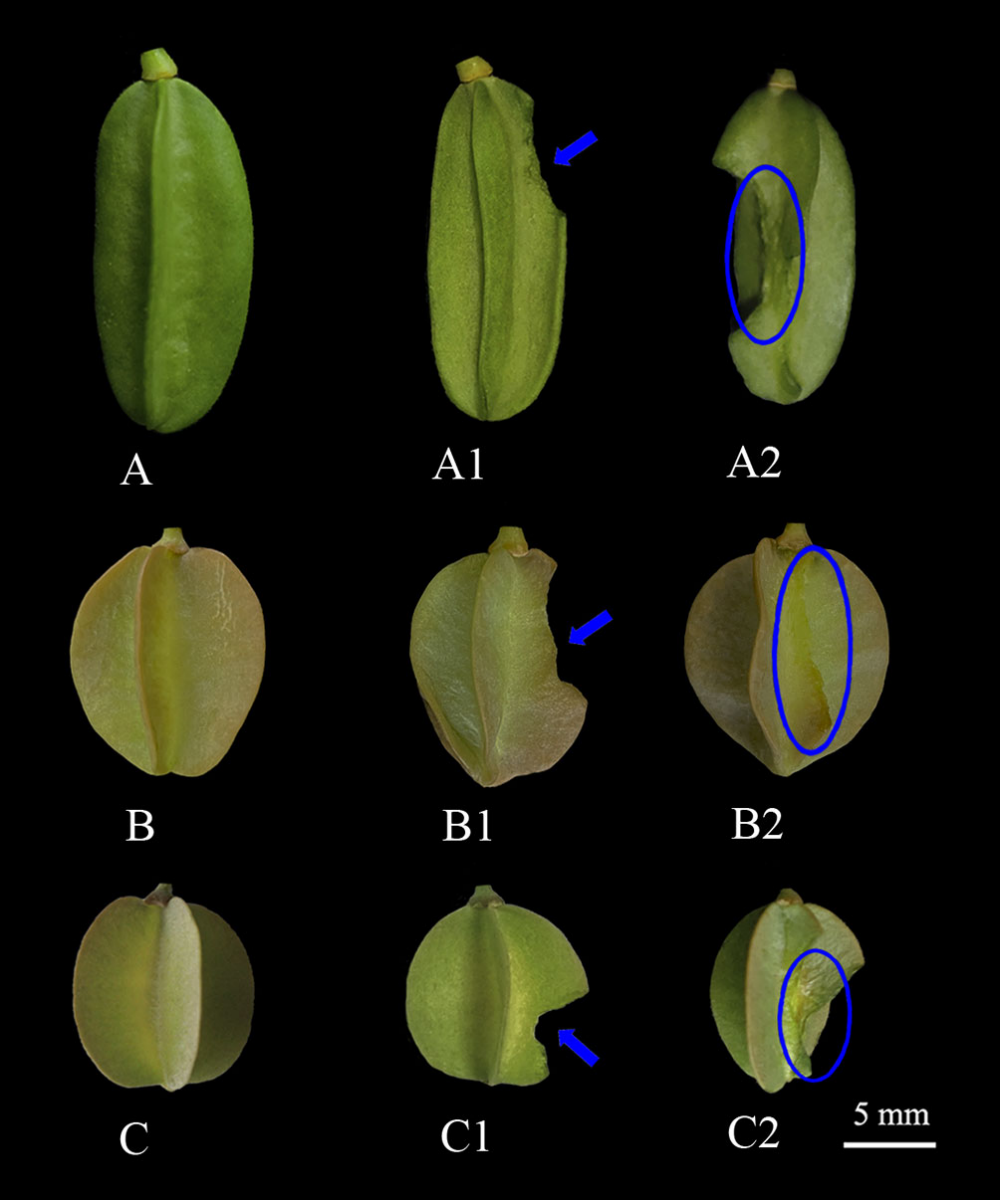
Winged fruits representing different insect herbivory categories of the three *Zygophyllum* species. (A, A1, A2) *Z. lehmannianum*; (B, B1, B2) *Z. potaninii*; (C, C1, C2) *Z. macropterum*; (A–C) intact (winged fruit with no damaged); (A1, B1, C1) low predation (winged fruit with damaged wings only); (A2, B2, C2) high predation (winged fruit with damaged both wings and fruit bodies).

Insect herbivory is very common during the development of winged fruits in natural populations of the three *Zygophyllum* species. In the 11 populations (**Supplementary Table 1**), three categories of winged fruits with insect herbivory (intact winged fruit, low predation, high predation) were identified (**Figure 1**). The mean percentage of winged fruit herbivory ranged from 16.37% to 31.19% across all study populations (**Table 1**). For *Z. potaninii* and *Z. macropterum*, the percentage of intact winged fruit category, low predation category, and high predation category had significant differences among the studied populations (all *P* < 0.001). For *Z. lehmannianum*, there was no significant difference in the percentage of insect herbivory categories among the studied populations (all *P* > 0.05) (**Table 1**). In the same population, the percentage of intact winged fruit was significantly higher than that of the other two categories (all *P* < 0.001). Moreover, the percentage of low predation was significantly higher than that of high predation (all *P* < 0.001) (**Table 1**).

**Table 1:** Percentage (mean ± SE) of winged fruits in different insect herbivory categories in 11 populations of the three *Zygophyllum* species.

Species	Population	Herbivory category (%)		
		Intact	Low predation	High predation
*Zygophyllum potaninii*	P1	68.81 ± 1.71^Aa^	25.17 ± 1.48^Ab^	6.02 ± 0.43^Ac^
	P2	72.10 ± 1.33^Ba^	22.82 ± 1.13^ABb^	5.09 ± 0.32^Ac^
	P3	72.39 ± 1.69^Ba^	21.99 ± 1.33^Bb^	5.62 ± 0.57^Ac^
	P4	76.98 ± 0.92^Ca^	18.46 ± 0.77^Cb^	4.56 ± 0.39^Bc^
*Z. macropterum*	P5	71.58 ± 0.88^Aa^	20.88 ± 0.71^Ab^	7.54 ± 0.41^Ac^
	P6	75.80 ± 0.62^Ba^	18.18 ± 0.61^Bb^	6.02 ± 0.22^Bc^
	P7	78.11 ± 0.62^Ba^	16.50 ± 0.48^Bb^	5.39 ± 0.27^Bc^
*Z. lehmannianum*	P8	81.67 ± 0.57^Aa^	13.94 ± 0.53^Ab^	4.39 ± 0.23^Ac^
	P9	81.81 ± 0.83^Aa^	13.69 ± 0.59^Ab^	4.50 ± 0.30^Ac^
	P10	82.87 ± 0.68^Aa^	13.13 ± 0.53^Ab^	4.00 ± 0.27^Ac^
	P11	83.63 ± 0.91^Aa^	12.56 ± 0.65^Ab^	3.81 ± 0.33^Ac^

Intact, winged fruit with no damaged; Low predation, winged fruit with damaged wings only; High predation, winged fruit with damaged both wings and fruit bodies. Different uppercase letters indicate significant differences among the study populations in the same insect herbivory category and different lowercase letters indicate significant differences among different insect herbivory categories in the same population (P < 0.05).

### 3.2 Effect of insect herbivory on seed set and seed mass

**Figure 2:**
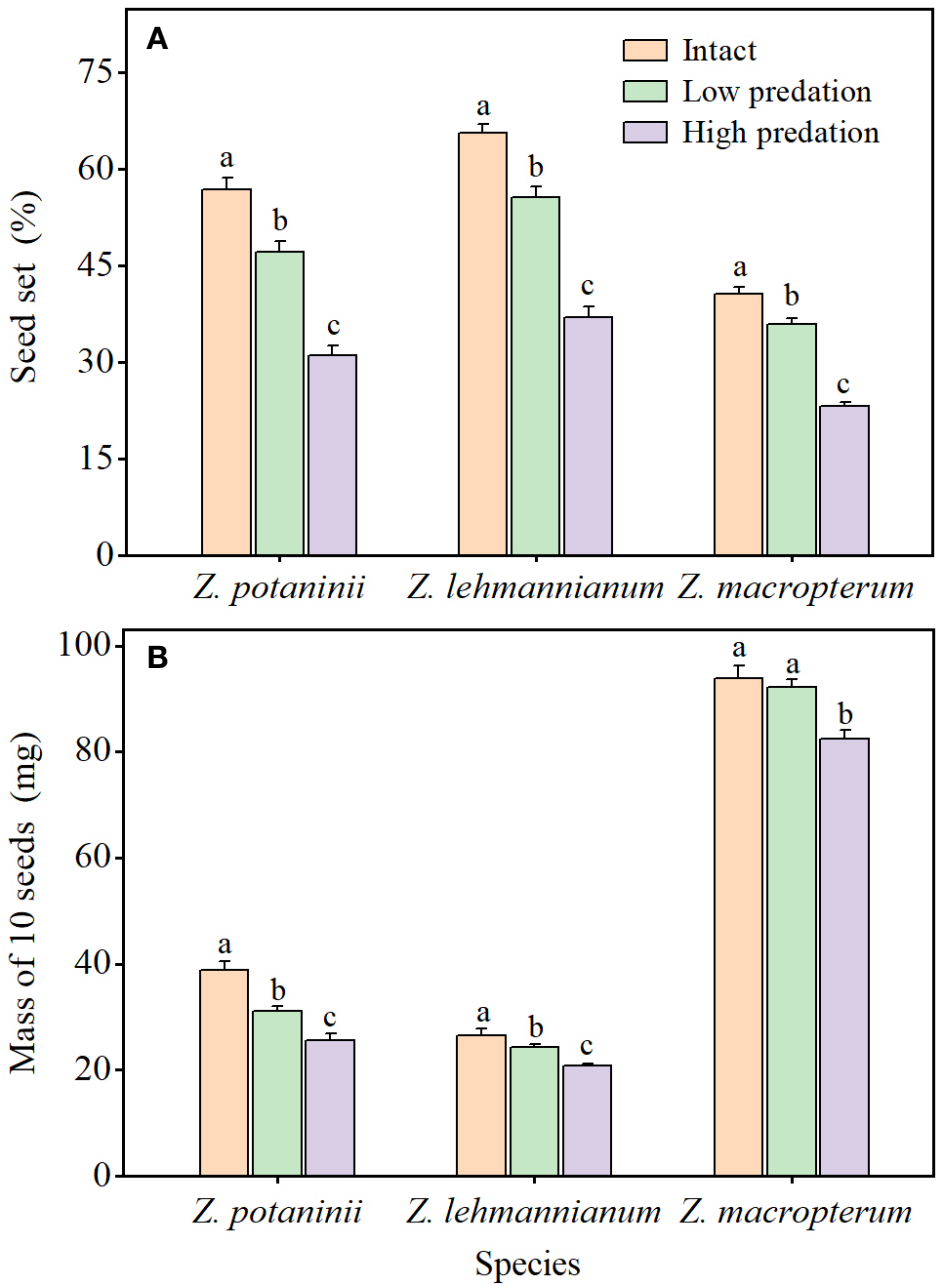
Effect of different insect herbivory categories on seed set **(A)** and seed mass **(B)** of the three *Zygophyllum* species (mean ± SE). Intact, winged fruit with no damaged; Low predation, winged fruit with damaged wings only; High predation, winged fruit with damaged both wings and fruit bodies. Bars with different lowercase letters indicate significant differences among different insect herbivory categories in the same species (*P* < 0.05).

For the three species, the seed set (**Figure 2A**) and seed mass (**Figure 2B**) exhibited the same trend across the three categories of insect herbivory: intact winged fruit > low predation > high predation. Insect herbivory had significant effects on seed set (*Z. potaninii*: *F* = 58.186, *P* < 0.001; *Z. lehmannianum*: *F* = 83.234, *P* < 0.001; *Z. macropterum*: *F* = 98.090, *P* < 0.001) and seed mass (*Z. potaninii*: *F* = 51.765, *P* < 0.001; *Z. lehmannianum*: *F* = 50.224, *P* < 0.001; *Z. macropterum*: *F* = 42.773, *P* < 0.001). For each species, the seed set of intact winged fruit was significantly higher than that of the other two categories (all *P* < 0.001), and low predation was significantly higher than that of high predation (all *P* < 0.001) (**Figure 2A**). The seed mass of intact winged fruit was significantly higher than that of the other two categories (all *P* < 0.001), except for *Z. macropterum*, where the seed mass of intact winged fruit was higher than that of low predation but not significantly (*P* = 0.235), and low predation was significantly higher than that of high predation (all *P* < 0.001) (**Figure 2B**).

### 3.3 Effect of insect herbivory on seed germination

For the three species, the germination percentages of freshly matured seeds were significantly affected by temperature (*Z. potaninii*: ***x*^2^** = 159.525, *P* < 0.001; *Z. lehmannianum*: ***x*^2^** = 194.357, *P* < 0.001; *Z. macropterum*: ***x*^2^** = 84.958, *P* < 0.001), light (*Z. potaninii*: ***x*^2^** = 81.373, *P* < 0.001; *Z. lehmannianum*: ***x*^2^** = 20.232, *P* < 0.001; *Z. macropterum*: ***x*^2^** = 106.506, *P* < 0.001) and their interaction (*Z. potaninii*: ***x*^2^** = 19.262, *P* < 0.05; *Z. lehmannianum*: ***x*^2^** = 34.258, *P* < 0.001; *Z. macropterum*: ***x*^2^** = 116.457, *P* < 0.001).

For *Z. potaninii*, the maximum germination percentages were observed at 35/20°C, with 66% and 85% under light and darkness, respectively, which were significantly higher than the percentages under other temperature regimes (all *P* < 0.05) (**Supplementary Figure 1A**). The optimum condition for germination was 35/20°C in darkness. For *Z. lehmannianum*, the maximum germination percentages were observed at 35/20°C, with 69% and 81% under light and darkness, respectively, which were significantly higher than those under other temperature regimes (all *P* < 0.05) (**Supplementary Figure 1B**). Consequently, the optimum condition for germination was 35/20°C in darkness. For *Z. macropterum*, the maximum germination percentage was 64% in darkness at 30/15°C and which was significantly higher than that under other temperature regimes (all *P* < 0.05) (**Supplementary Figure 1C**). The germination was significantly higher in darkness compared to light at all temperature regimes (all *P* < 0.05), except at 5/2°C and 40/25°C (**Supplementary Figure 1C**), with the optimum condition for germination was 30/15°C in darkness.

**Figure 3:**
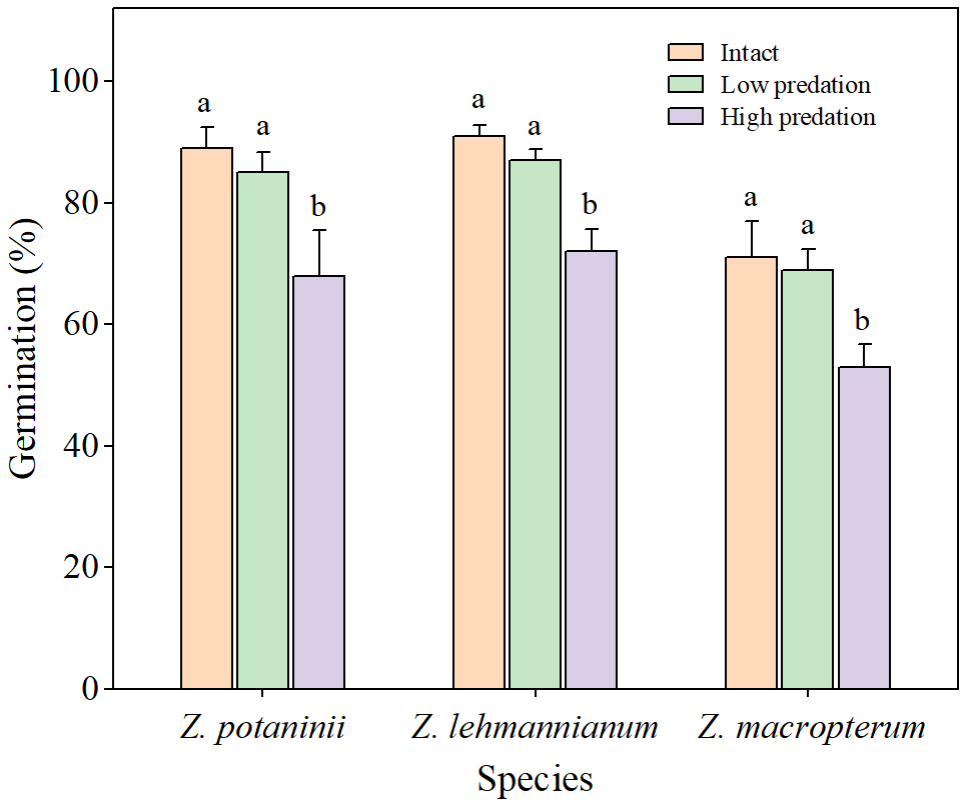
Germination percentages of the seeds from winged fruits in different insect herbivory categories at the optimum conditions of the three *Zygophyllum* species (mean ± SE). Intact, winged fruit with no damaged; Low predation, winged fruit with damaged wings only; High predation, winged fruit with damaged both wings and fruit bodies. Bars with different lowercase letters indicate significant differences among different insect herbivory categories in the same species (*P* < 0.05).

Under these optimum conditions, there were significant differences in the germination percentages of seeds from winged fruits among the different insect herbivory categories (*Z. potaninii*: ***x*^2^** = 13.722, *P* < 0.05; *Z. lehmannianum*: ***x*^2^** = 13.462, *P* < 0.05; *Z. macropterum*: ***x*^2^** = 8.351, *P* < 0.05). For the three species, germination percentages of intact winged fruit and low predation were significantly higher than those of high predation (all *P* < 0.05) (**Figure 3**). Although the germination percentage of intact winged fruit was higher than that of low predation, the difference was not significant (all *P* > 0.05) (**Figure 3**).

### 3.4 Effect of insect herbivory on seedling growth

**Figure 4:**
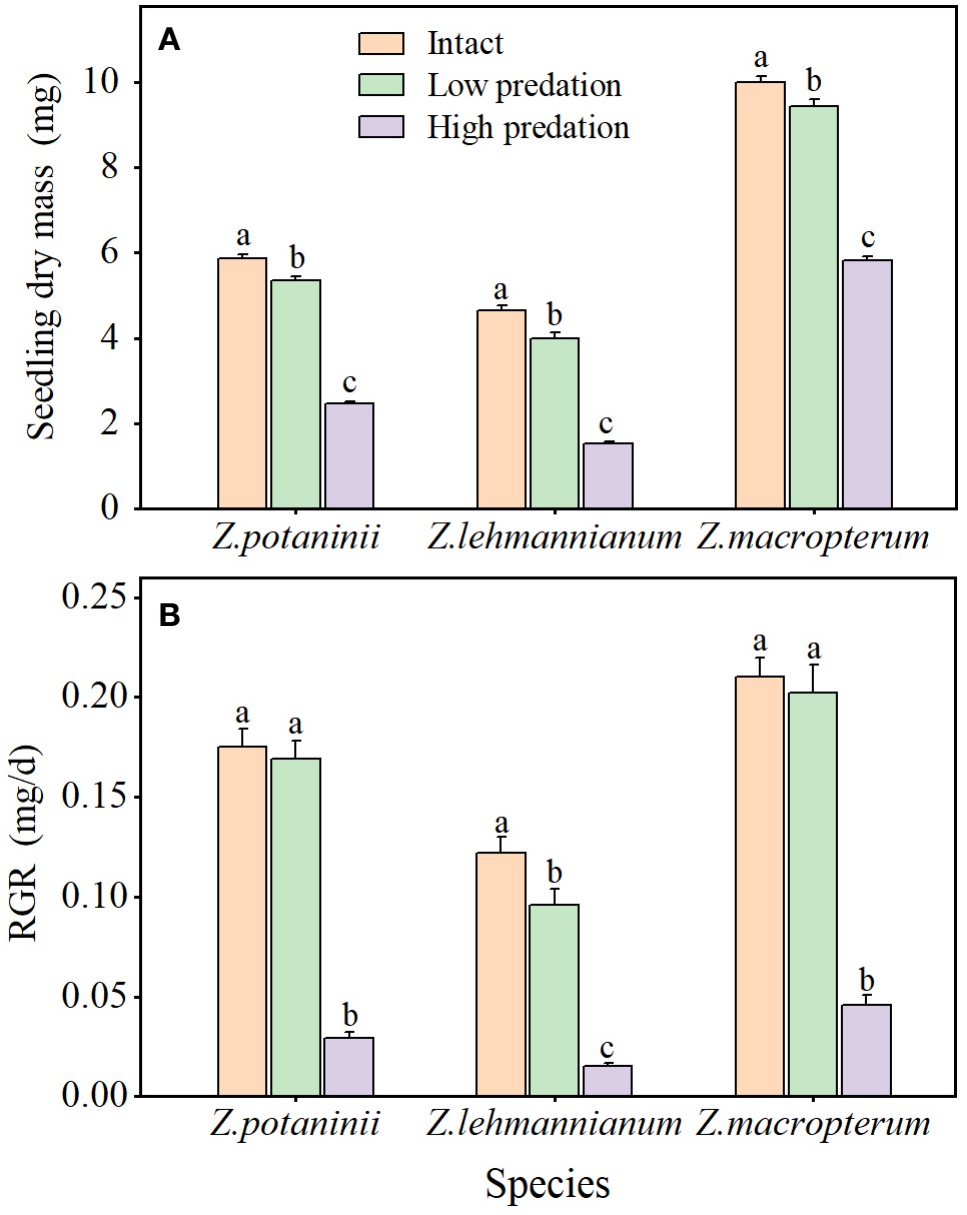
Effect of different insect herbivory categories on seedling dry mass **(A)** and relative growth rate (RGR) **(B)** of the three *Zygophyllum* species (mean ± SE). Intact, winged fruit with no damaged; Low predation, winged fruit with damaged wings only; High predation, winged fruit with damaged both wings and fruit bodies. Bars with different lowercase letters indicate significant differences among different insect herbivory categories in the same species (*P* < 0.05).

For the three species, insect herbivory had significant effects on seedling dry mass (*Z. potaninii*: *F* = 622.864, *P* < 0.001; *Z. lehmannianum*: *F* = 527.105, *P* < 0.001; *Z. macropterum*: *F* = 300.859, *P* < 0.001) and seedling RGR (*Z. potaninii*: *F* = 130.191, *P* < 0.001; *Z. lehmannianum*: *F* = 219.858, *P* < 0.001; *Z. macropterum*: *F* = 77.790, *P* < 0.001). Seedling dry mass of intact winged fruit was significantly higher than that of the other two categories (all *P* < 0.05), and low predation was significantly higher than that of high predation (all *P* < 0.001) (**Figure 4A**). Seedling RGR of intact winged fruit and low predation were significantly higher than those of high predation (all *P* < 0.001) (**Figure 4B**). For *Z. lehmannianum*, seedling RGR of intact winged fruit was significantly higher than that of low predation, while for *Z. potaninii* (*P* = 0.543) and *Z. macropterum* (*P* = 0.599), intact winged fruit were higher than those of low predation but not significantly (**Figure 4B**). Furthermore, both seedling dry mass and RGR exhibited a decrease as the degree of insect herbivory increased (**Figures 4A, B**).

## 4 Discussion

Fruit predation can significantly reduce the reproductive potential of plants (Kolb et al., 2008). The impact of fruit predation on plant populations depends on predation rates and whether these rates vary predictably across species and environments (Palmisano and Fox, 1997; Maron and Crone, 2006; Romera et al., 2013). In our study, the percentage of winged fruits damaged by insects ranged from 16 to 31% across 11 populations of the three *Zygophyllum* species in the Junggar Desert of Xinjiang, China. Previous studies have shown wide variation in fruit predation by insects among different species, such as 60% fruit predation in *Attalea phalerata* (Quiroga-Castro and Roldán, 2001) and 15% in *Attalea vitrivir* (Ferreira et al., 2016). In addition, insect herbivory of *Astragalus lehmannianus* in a similar desert in northern Xinjiang was found to be 16-22% (Han et al., 2018). These variations in insect herbivory may be influenced by a number of factors including plant phenology (Gherlenda et al., 2016), large mammalian herbivores (Ohgushi, 2005; Berman et al., 2019), temperature (Lemoine et al., 2014), and light availability (Suárez-Vidal et al., 2017). In addition, previous studies have suggested that the fleshy defense layer of fruit may serve as a barrier to insect predators or provide ecological resources that help reduce damage to the seeds (Mack, 2000). In our study, for the three species, the percentage of winged fruits with damaged wings was significantly higher than those with damaged both wings and fruit bodies. This may suggest that the fleshy fruit wings act as a barrier to insects or provide them with nutrition, thereby reducing damage to the fruit and seeds caused by insect herbivory.

Insect herbivory can affect the development of undamaged seeds co-occurring in predated fruits as a result of the plant’s response to the attack (Bonal et al., 2007). Selective seed abortion in response to insect herbivory is likely to occur in fruits with multiple seeds (Meyer et al., 2014). One possibility is that plants allocate fewer resources to predated fruits to reduce the availability of food for predators, resulting in co-occurring seeds facing growth rate constraints and a high probability of abortion (Fernandes and Whitham, 1989; Verdú and García-Fayos, 1998). Furthermore, the plant may reduce the resources allocated to predated fruits, leading to a reduction in the size of the remaining undamaged co-occurring seeds (Verdú and García-Fayos, 1998; DeSoto et al., 2016). In our study, for the three species, the seed set and seed mass of winged fruits with different insect herbivory categories (low and high predation) were significantly lower than those of intact winged fruit. This indicates a negative effect of insect herbivory on seed development, which is consistent with findings on *Aloe pretoriensis* where the seed set of predated fruits was considerably reduced, resulting in an average seed loss of up to 62% (Kaylee et al., 2019).

Additionally, the seed set and seed mass decreased for each species with increasing intensity of insect herbivory. Although the seed set and seed mass of winged fruits with low predation were lower than those of intact winged fruit, they were still significantly higher than those with high predation. This indicates that the negative effect of low predation on seed development was significantly less than that of high predation. These results suggest that the presence of fleshy fruit wings alleviates the effect of insect herbivory on seed development by reducing insect herbivory on the fruit body. A similar protective mechanism has been found in *Guazuma ulmifolia*, where the well-developed fleshy tissue that acts as a barrier or reward for predators, thus reducing damage to the seed (Herrera, 1989; Mack, 2000).

Understanding the relationship between fruit predation and seed germination is of significant importance as it is closely related to the reproductive success of the plant (Alencar et al., 2012). Fruit predation typically has negative effects on plants by reducing seed germination and seedling establishment (Janzen, 1971; Kolb et al., 2008). In our study, the germination percentages of the three species decreased with increasing intensity of fruit predation. However, seed germination percentage of winged fruit with low predation did not differ from intact winged fruit, whereas it was significantly lower in winged fruits with high predation compared to intact and low predation winged fruits. This indicates that insect herbivory on fruit wings has no effect on seed germination, but when both wings and fruit bodies are damaged, seed germination is impeded. This may be because insect herbivory on both wings and fruit bodies significantly affects seed development, resulting in a significant decrease in seed germination. A previous study has shown that the germination percentage of heavy-predation seeds was significantly lower than that of low-predation and medium-predation seeds (Han et al., 2018). Also, previous studies have shown that seeds from damaged fruits have significantly lower germination percentage compared to undamaged fruits in *Mimosa bimucronata* (Tomaz et al., 2007) and *Juniperus thurifera* (DeSoto et al., 2016). In contrast, for the three *Zygophyllum* species, the seed germination percentage of winged fruits with damaged both wings and fruit bodies was significantly lower than that of winged fruits with damaged wings only. A possible explanation is that insect damage exceeds the protective threshold of fruit wings, rendering the protective mechanism ineffective (Lemoine et al., 2017).

The period between seed germination and early seedling establishment is one of the most vulnerable stages in the life cycle of plants in desert environments (Zhang et al., 2021). In our study of the three *Zygophyllum* species, the seedling dry mass and RGR of winged fruits with two categories of insect herbivory were lower than those of intact winged fruit. Moreover, the seedling dry mass and RGR of winged fruits with damaged wings were significantly higher than those of winged fruits with damaged both wings and fruit bodies. This indicates that seedling growth was significantly affected by high levels of insect herbivory. Previous studies have shown that fruit predation by insects results in decreased seedling growth, suggesting that the effects of insect predation extend to the offspring of the attacked species (Mueller et al., 2005). These results suggest that the presence of fruit wings may reduce insect herbivory on fruit bodies, thus lessening the detrimental effect on seedling growth. Consequently, the fleshy fruit wings of the three *Zygophyllum* species may provide protection for the seeds to alleviate harm from insect predation before dispersal, thereby improving the fitness of the seeds.

## 5 Conclusion

For the three *Zygophyllum* species, the seed set, seed mass, seed germination, seedling dry mass, and seedling relative growth rate (RGR) exhibited the same trend across the three categories of insect herbivory: intact winged fruit > low predation > high predation. In particular, winged fruits with damaged wings only (low predation) were all significantly higher than those of winged fruits with damaged both wings and fruit bodies (high predation). Our study suggests that the presence of the fruit wings may provide protection for the seeds to alleviate harm from insect predation before dispersal. This “protective mechanism” may be an adaptation of the plant to insect herbivory in the environment. Consequently, further exploration of the ecological function of the fruit wings is warranted to improve current understanding of the evolution of selective pressures on winged fruits.

## Data availability statement

The original contributions presented in the study are included in the article/**Supplementary Material**. Further inquiries can be directed to the corresponding author.

## Author contributions

KX: Data curation, Investigation, Methodology, Writing – original draft. JQ: Investigation, Methodology, Writing – original draft. JM: Data curation, Formal Analysis, Writing – original draft. YL: Investigation, Methodology, Writing – original draft. DT: Project administration, Visualization, Writing – review & editing.
