# Supplementary material for: Protection against insect predation during fruit development: the role of fleshy fruit wings of three species of Zygophyllum in the cold desert of Central Asia
Source: Front Plant Sci. 2023 Nov 14;14:1267714. doi: 10.3389/fpls.2023.1267714 (PMC10682714; doi:10.3389/fpls.2023.1267714)
Supplement: Supplementary file 1 [file DataSheet_1.docx]

Supplementary Material

Protection against insect predation during fruit development: the role of fleshy fruit wings of three species of Zygophyllum in the cold desert of Central Asia

**Kaiqing Xie^1,2^, Juan Qiu^2^, Jannathan Mamut^1,2^, Yuting Li^2^ and Dunyan Tan^2*^**

*** Correspondence:** Dunyan Tan: [tandunyan@163.com](mailto:tandunyan@163.com)

#
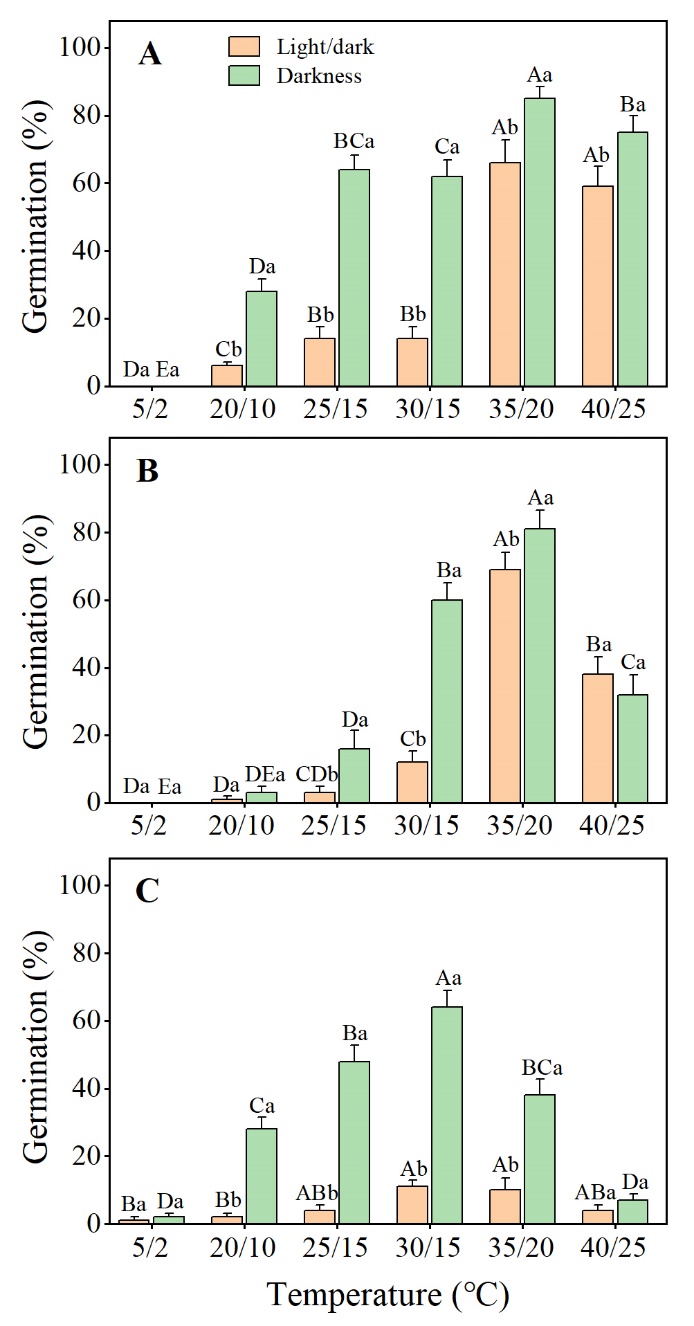
Supplementary Figure

## Supplementary Figure 1. Germination percentages of fresh matured seeds in light and in darkness at six temperature regimes of the three *Zygophyllum* species (mean ± SE). (A) *Z. potaninii*, (B) *Z. lehmannianum*, (C) *Z. macropterum*. Bars with different uppercase letters indicate significant differences among different temperature regimes in light or in darkness and different lowercase letters indicate significant differences between in light and in darkness at the same temperature (*P* < 0.05).
