# Supplementary material for: Protection against insect predation during fruit development: the role of fleshy fruit wings of three species of Zygophyllum in the cold desert of Central Asia
Source: Front Plant Sci. 2023 Nov 14;14:1267714. doi: 10.3389/fpls.2023.1267714 (PMC10682714; doi:10.3389/fpls.2023.1267714)
Supplement: Supplementary file 2 [file Table_1.docx]

Supplementary Material

Protection against insect predation during fruit development: the role of fleshy fruit wings of three species of Zygophyllum in the cold desert of Central Asia

**Kaiqing Xie^1,2^, Juan Qiu^2^, Jannathan Mamut^1,2^, Yuting Li^2^ and Dunyan Tan^2*^**

*** Correspondence:** Dunyan Tan: [tandunyan@163.com](mailto:tandunyan@163.com)

# Supplementary Table

**Supplementary Table 1.** Location of the study populations of three *Zygophyllum* species in the Junggar Desert of Xinjiang, China.

| **Species** | **Population** | **Latitude (N)** | **Longitude (E)** | **Altitude (m)** |
| --- | --- | --- | --- | --- |
| *Zygophyllum potaninii* | P1 | 45°03′ | 89°13′ | 907 |
|  | P2 | 45°06′ | 89°17′ | 984 |
|  | P3 | 45°29′ | 89°29′ | 1052 |
|  | P4 | 45°48′ | 84°51′ | 815 |
| *Z. macropterum* | P5 | 45°10′ | 89°22′ | 1097 |
|  | P6 | 44°11′ | 86°06′ | 817 |
|  | P7 | 43°48′ | 87°34′ | 912 |
| *Z. lehmannianum* | P8 | 44°23′ | 87°53′ | 452 |
|  | P9 | 44°57′ | 88°52′ | 518 |
|  | P10 | 45°28′ | 86°25′ | 323 |
|  | P11 | 44°11′ | 86°06′ | 804 |
